# Supplementary material for: Evolution of the gene regulatory network of body axis by enhancer hijacking in amphioxus
Source: eLife. 2024 Jan 17;13:e89615. doi: 10.7554/eLife.89615 (PMC10794066; doi:10.7554/eLife.89615)
Supplement: Supplementary file 1. — (a) The expression pattern of Nodal gene in representative deuterostomes; (b) the expression pattern of Gdf1/3 and its paralogs in representative deuterostomes. [file elife-89615-supp1.docx]

**Supplementary file 1a**

The expression pattern of *Nodal* gene in representative deuterostomes

| **Taxa** | **Species** | **Gene name/alias** | **Maternal** | **Zygotic** | **unilateral** | **References** |
| --- | --- | --- | --- | --- | --- | --- |
| **Echinoderms** | *Strongylocentrotus purpuratus* | *Nodal* | no | yes | Yes | (*Lapraz et al., 2006*) |
| **Hemichordates** | *Ptychodera flava* | *Nodal* | no | yes | n.d. | (*Rottinger et al., 2015*) |
| **Cephalochordates** | *Branchiostoma floridae* | *Nodal* | yes | yes | yes | (*Onai et al., 2010*)  (*Soukup et al., 2015*) |
| **Urochordates** | *Ciona intestinalis* | *Nodal* | no | yes | yes | (*Hudson and Yasuo, 2005*)  (*Yoshida and Saiga, 2008*) |
| **Actinopterygians** | *Polypterus senegalus*  (bichir) | *Sqt* | no | yes | n.d. | (*Takeuchi et al., 2009*) |
|  |  | *Cyc* | no | yes | n.d. | (*Takeuchi et al., 2009*) |
|  | *Danio rerio*  (teleost) | *Ndr1*/*Squint*/*Sqt* | yes | yes | no | (*Rebagliati et al., 1998*) |
|  |  | *Ndr2*/*Cyclops*/*Cyc* | no | yes | yes | (*Rebagliati et al., 1998*) |
|  |  | *Ndr3*/*Southpaw*/*Spw* | no | yes | yes | (*Long et al., 2003*) |
| **Frogs** | *Xenopus laevis* | *Xnr1* | no | yes | yes | (*Jones et al., 1995*)  (*Lowe et al., 1996*) |
|  |  | *Xnr2* | no | yes | no | (*Jones et al., 1995*) |
|  |  | *Xnr3* | no | yes | no | (*Smith et al., 1995*) |
|  |  | *Xnr4* | no | yes | no | (*Joseph and Melton, 1997*) |
|  |  | *Xnr5* | no | yes | no | (*Takahashi et al., 2000*)  (*Tadjuidje et al., 2016*) |
|  |  | *Xnr6* | no | yes | no | (*Takahashi et al., 2000*) |
| **Mammals** | *Mus musculus* | *Nodal* | n.d. | yes | yes | (*Zhou et al., 1993*)  (*Lowe et al., 1996*) |
| **Birds** | *Gallus gallus* | *cNR1* | n.d. | yes | yes | (*Levin et al., 1995*)  (*Chapman et al., 2002*) |

n.d., not determined. Only one copy of *Nodal* gene presents in invertebrate lineages. The phylogeny of vertebrate *Nodal* paralogues was analyzed previously (*Opazo et al., 2019*). At early stage, all analyzed deuterostome *Nodal* genes are not maternally supplied except the *Nodal* of cephalochordate amphioxus and *Ndr1*/*Squint*/*Sqt* of zebrafish. Both *Sqt* and *Cyc* in bichir (a stem group of actinopterygii) are expressed zygotically only at early blastula, suggesting that the maternal expression of *Sqt* in zebrafish might be a lineage-specific diversification in teleost (a derived group of actinopterygii) (*Takeuchi et al., 2009*). In addition, the maternal *Sqt* RNA functions independently of Nodal signaling but as a noncoding scaffold (*Lim et al., 2012*). At neurula stage of chordate, at least one *Nodal* gene, which is involved in L-R axis patterning, is expressed unilaterally (left side). Similarly, in echinoderm larva, *Nodal* is expressed unilaterally (right side).

**Supplementary file 1b**

The expression pattern of *Gdf1/3* and its paralogs in representative deuterostomes

| **Taxa** | **Species** | **Gene name/alias** | **Maternal** | **Zygotic** | **Unilateral** | **References** |
| --- | --- | --- | --- | --- | --- | --- |
| **Echinoderms** | *Strongylocentrotus purpuratus* | *Univin* | yes | yes | no | (*Lapraz et al., 2006*)  (*Bessodes et al., 2012*) |
| **Cephalochordate** | *Branchiostoma floridae* | *GDF1/3-like1*/*Vg1* | yes | yes | yes | (*Onai et al., 2010*)  (*Soukup et al., 2015*) |
| **Actinopterygians** | *Danio rerio* | *Gdf3/Vg1*/*Dvr1* | yes | yes | no | (*Pelliccia et al., 2017*) |
| **Frogs** | *Xenopus laevis* | *Vg1*/*Gdf1_A_* | yes | n.d. | n.d. | (*Weeks and Melton, 1987*)  (*Opazo et al., 2019*) |
|  |  | *Derrière*/*Gdf3_A_* | no | yes | no | (*Sun et al., 1999*)  (*Hanafusa et al., 2000*)  (*Opazo et al., 2019*) |
| **Mammals** | *Mus musculus* | *Gdf1*/*Gdf1_M_* | n.d. | yes | no | (*Wall et al., 2000*)  (*Rankin et al., 2000*) |
|  |  | *Gdf3* /*Gdf3_M_* | n.d. | yes | no | (*Chen et al., 2006*) |
| **Birds** | *Gallus gallus* | *cVg1* | n.d. | yes | no | (*Seleiro et al., 1996*)  (*Somi et al., 2003*) |

n.d., not determined. At least one *Gdf1/3* gene in deuterostome lineages analyzed is expressed maternally. The *Gdf1/3* gene was duplicated independently in anura and mammalian within their own lineage (*Opazo and Zavala, 2018; Opazo et al., 2019*). The A and M subscripts were used for the genes in anura (frogs) and mammalian respectively (*Opazo et al., 2019*). At neurula stage of vertebrates, all *Gdf1/3* genes are expressed bilaterally. In echinoderm larva, the *Gdf1/3* gene is expressed bilaterally also (though left-right asymmetrically) (*Bessodes et al., 2012*). However, in amphioxus, one of *Gdf1/3* genes analyzed is expressed in the left side only. Note that our new data of WISH and reexamination of transcriptome data could not detect any maternal expression of either of the two *Gdf1/3* copies in Florida amphioxus.

**Supplemental references**

Bessodes, N., E. Haillot, V. Duboc, E. Rottinger, F. Lahaye and T. Lepage (2012). "Reciprocal signaling between the ectoderm and a mesendodermal left-right organizer directs left-right determination in the sea urchin embryo." *PLoS Genet* **8**(12): e1003121.

Chapman, S. C., F. R. Schubert, G. C. Schoenwolf and A. Lumsden (2002). "Analysis of spatial and temporal gene expression patterns in blastula and gastrula stage chick embryos." *Dev Biol* **245**(1): 187-199.

Chen, C., S. M. Ware, A. Sato, D. E. Houston-Hawkins, R. Habas, M. M. Matzuk, M. M. Shen and C. W. Brown (2006). "The Vg1-related protein Gdf3 acts in a Nodal signaling pathway in the pre-gastrulation mouse embryo." *Development* **133**(2): 319-329.

Hanafusa, H., N. Masuyama, M. Kusakabe, H. Shibuya and E. Nishida (2000). "The TGF-β family member derrière is involved in regulation of the establishment of left–right asymmetry." *EMBO reports* **1**(1): 32-39.

Hudson, C. and H. Yasuo (2005). "Patterning across the ascidian neural plate by lateral Nodal signalling sources."

Jones, C. M., M. R. Kuehn, B. Hogan, J. C. Smith and C. Wright (1995). "Nodal-related signals induce axial mesoderm and dorsalize mesoderm during gastrulation." *Development* **121**(11): 3651-3662.

Joseph, E. M. and D. A. Melton (1997). "Xnr4: AXenopusNodal-Related Gene Expressed in the Spemann Organizer." *Developmental biology* **184**(2): 367-372.

Lapraz, F., E. Rottinger, V. Duboc, R. Range, L. Duloquin, K. Walton, S. Y. Wu, C. Bradham, M. A. Loza, T. Hibino, K. Wilson, A. Poustka, D. McClay, L. Angerer, C. Gache and T. Lepage (2006). "RTK and TGF-beta signaling pathways genes in the sea urchin genome." *Dev Biol* **300**(1): 132-152.

Levin, M., R. L. Johnson, C. D. Sterna, M. Kuehn and C. Tabin (1995). "A molecular pathway determining left-right asymmetry in chick embryogenesis." *Cell* **82**(5): 803-814.

Lim, S., P. Kumari, P. Gilligan, H. N. Quach, S. Mathavan and K. Sampath (2012). "Dorsal activity of maternal squint is mediated by a non-coding function of the RNA." *Development* **139**(16): 2903-2915.

Long, S., N. Ahmad and M. Rebagliati (2003). "The zebrafish nodal-related gene southpaw is required for visceral and diencephalic left-right asymmetry." *Development* **130**(11): 2303-2316.

Lowe, L. A., D. M. Supp, K. Sampath, T. Yokoyama, C. V. Wright, S. S. Potter, P. Overbeek and M. R. Kuehn (1996). "Conserved left–right asymmetry of nodal expression and alterations in murine situs inversus." *Nature* **381**(6578): 158-161.

Onai, T., J. K. Yu, I. L. Blitz, K. W. Cho and L. Z. Holland (2010). "Opposing Nodal/Vg1 and BMP signals mediate axial patterning in embryos of the basal chordate amphioxus." *Dev Biol* **344**(1): 377-389.

Opazo, J. C., S. Kuraku, K. Zavala, J. Toloza-Villalobos and F. G. Hoffmann (2019). "Evolution of nodal and nodal-related genes and the putative composition of the heterodimers that trigger the nodal pathway in vertebrates." *Evol Dev* **21**(4): 205-217.

Opazo, J. C. and K. Zavala (2018). "Phylogenetic evidence for independent origins of GDF1 and GDF3 genes in anurans and mammals." *Sci Rep* **8**(1): 13595.

Pelliccia, J. L., G. A. Jindal and R. D. Burdine (2017). "Gdf3 is required for robust Nodal signaling during germ layer formation and left-right patterning." *Elife* **6**.

Rankin, C. T., T. Bunton, A. M. Lawler and S.-J. Lee (2000). "Regulation of left-right patterning in mice by growth/differentiation factor-1." *Nature genetics* **24**(3): 262-265.

Rebagliati, M. R., R. Toyama, C. Fricke, P. Haffter and I. B. Dawid (1998). "Zebrafish nodal-related genes are implicated in axial patterning and establishing left–right asymmetry." *Developmental biology* **199**(2): 261-272.

Rottinger, E., T. Q. DuBuc, A. R. Amiel and M. Q. Martindale (2015). "Nodal signaling is required for mesodermal and ventral but not for dorsal fates in the indirect developing hemichordate, Ptychodera flava." *Biol Open* **4**(7): 830-842.

Seleiro, E. A., D. J. Connolly and J. Cooke (1996). "Early developmental expression and experimental axis determination by the chicken Vg1 gene." *Current Biology* **6**(11): 1476-1486.

Smith, W. C., R. McKendry, S. Ribisi Jr and R. M. Harland (1995). "A nodal-related gene defines a physical and functional domain within the Spemann organizer." *Cell* **82**(1): 37-46.

Somi, S., A. C. Houweling, A. A. Buffing, A. F. Moorman and M. J. Van Den Hoff (2003). "Expression of cVg1 mRNA during chicken embryonic development." *The Anatomical Record Part A: Discoveries in Molecular, Cellular, and Evolutionary Biology: An Official Publication of the American Association of Anatomists* **273**(1): 603-608.

Soukup, V., L. W. Yong, T.-M. Lu, S.-W. Huang, Z. Kozmik and J.-K. Yu (2015). "The Nodal signaling pathway controls left-right asymmetric development in amphioxus." *EvoDevo* **6**(1): 1-23.

Sun, B. I., S. M. Bush, L. A. Collins-Racie, E. R. LaVallie, E. A. DiBlasio-Smith, N. M. Wolfman, J. M. McCoy and H. L. Sive (1999). "derriere: a TGF-beta family member required for posterior development in Xenopus." *Development* **126**(7): 1467-1482.

Tadjuidje, E., M. Kofron, A. Mir, C. Wylie, J. Heasman and S. W. Cha (2016). "Nodal signalling in Xenopus: the role of Xnr5 in left/right asymmetry and heart development." *Open Biol* **6**(8).

Takahashi, S., C. Yokota, K. Takano, K. Tanegashima, Y. Onuma, J. Goto and M. Asashima (2000). "Two novel nodal-related genes initiate early inductive events in Xenopus Nieuwkoop center." *Development* **127**(24): 5319-5329.

Takeuchi, M., M. Takahashi, M. Okabe and S. Aizawa (2009). "Germ layer patterning in bichir and lamprey; an insight into its evolution in vertebrates." *Dev Biol* **332**(1): 90-102.

Wall, N. A., E. J. Craig, P. A. Labosky and D. S. Kessler (2000). "Mesendoderm induction and reversal of left-right pattern by mouse Gdf1, a Vg1-related gene." *Dev Biol* **227**(2): 495-509.

Weeks, D. and D. Melton (1987). "A maternal mRNA localized to the vegetal hemisphere in Xenopus eggs codes for a growth factor related to TGF-β." *Cell* **51**(5): 861-867.

Yoshida, K. and H. Saiga (2008). "Left-right asymmetric expression of Pitx is regulated by the asymmetric Nodal signaling through an intronic enhancer in Ciona intestinalis." *Dev Genes Evol* **218**(7): 353-360.

Zhou, X., H. Sasaki, L. Lowe, B. L. Hogan and M. R. Kuehn (1993). "Nodal is a novel TGF-β-like gene expressed in the mouse node during gastrulation." *Nature* **361**(6412): 543-547.
